# Iron Overload and Breast Cancer: Iron Chelation as a Potential Therapeutic Approach

**DOI:** 10.3390/life12070963

**Published:** 2022-06-27

**Authors:** Sufia Islam, Nazia Hoque, Nishat Nasrin, Mehnaz Hossain, Farhana Rizwan, Kushal Biswas, Muhammad Asaduzzaman, Sabera Rahman, David W. Hoskin, Saki Sultana, Christian Lehmann

**Affiliations:** 1 Department of Pharmacy, East West University, A/2, Jahurul Islam Avenue, Jahurul Islam City, Aftabnagar, Dhaka 1212, Bangladesh; nzh@ewubd.edu (N.H.); nishat@ewubd.edu (N.N.); frezwan@ewubd.edu (F.R.); ksb@ewubd.edu (K.B.); 2 Department of Political Science and Global Governance, Balsillie School of International Affairs, University of Waterloo, Waterloo, ON N2L 3G1, Canada; mhossain@balsillieschool.ca; 3 Department of Clinical Pharmacy and Pharmacology, University of Dhaka, Dhaka 1000, Bangladesh; asaduzzaman@du.ac.bd; 4 Department of Pharmacy, City University, Dhaka 1215, Bangladesh; sabera.pharmacy@cityuniversity.edu.bd; 5 Department of Pathology, Faculty of Medicine, Dalhousie University, Halifax, NS B3H 4R2, Canada; d.w.hoskin@dal.ca; 6 Department of Anesthesia, Pain Management and Perioperative Medicine, Faculty of Medicine, Dalhousie University, Halifax, NS B3H 4R2, Canada; saki.sultana@dal.ca (S.S.); chlehmann@dal.ca (C.L.)

**Keywords:** breast cancer, oxidative stress, iron overload, iron chelator, estrogen

## Abstract

Breast cancer has historically been one of the leading causes of death for women worldwide. As of 2020, breast cancer was reported to have overtaken lung cancer as the most common type of cancer globally, representing an estimated 11.3% of all cancer diagnoses. A multidisciplinary approach is taken for the diagnosis and treatment of breast cancer that includes conventional and targeted treatments. However, current therapeutic approaches to treating breast cancer have limitations, necessitating the search for new treatment options. Cancer cells require adequate iron for their continuous and rapid proliferation. Excess iron saturates the iron-binding capacity of transferrin, resulting in non-transferrin-bound iron (NTBI) that can catalyze free-radical reactions and may lead to oxidant-mediated breast carcinogenesis. Moreover, excess iron and the disruption of iron metabolism by local estrogen in the breast leads to the generation of reactive oxygen species (ROS). Therefore, iron concentration reduction using an iron chelator can be a novel therapeutic strategy for countering breast cancer development and progression. This review focuses on the use of iron chelators to deplete iron levels in tumor cells, specifically in the breast, thereby preventing the generation of free radicals. The inhibition of DNA synthesis and promotion of cancer cell apoptosis are the targets of breast cancer treatment, which can be achieved by restricting the iron environment in the body. We hypothesize that the usage of iron chelators has the therapeutic potential to control intracellular iron levels and inhibit the breast tumor growth. In clinical settings, iron chelators can be used to reduce cancer cell growth and thus reduce the morbidity and mortality in breast cancer patients.

## 1. Introduction

### 1.1. Importance of Iron in Human Health

**Figure 1:**
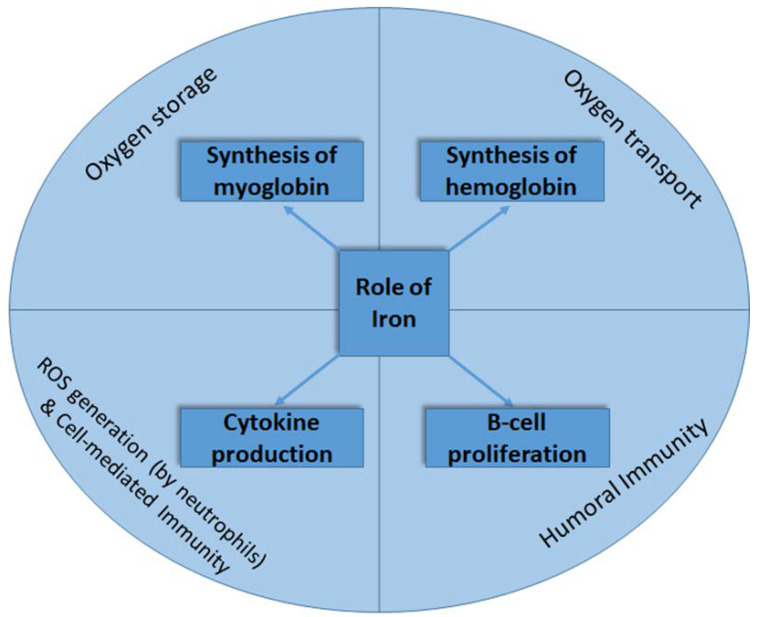
The roles of iron in oxygen transport and storage and in immune response generation.

Iron is a trace element that is essential for its important physiological roles in regulating the biochemical pathways of different cellular processes [1,2,3], including the oxidative response of neutrophils [4], cytokine production as part of cell-mediated immunity, the proliferation of B-lymphocytes, and the generation of humoral immune responses [2] (Figure 1). Almost two-thirds of the iron in the body is found in the hemoglobin present in erythrocytes. The remaining iron in the body is bound to the myoglobin in muscle tissue, as well as different iron-containing enzymes [5]. 

Usually, iron concentration is stabilized at the physiological level by a liver-derived hormone called hepcidin. When iron concentration is increased in the plasma, there is increased release of hepcidin. This, in turn, brings down the iron level by reducing its release from the iron storage sites by promoting the degradation of the iron exporter protein ferroportin [6]. Similarly, when iron concentration is decreased for any reason, hepcidin expression is reduced. Consequently, iron absorption is restored, resulting in increased iron levels in the plasma. It is established that iron deficiency or the accumulation of excess iron has significant health effects. Hypoferraemic conditions induced by infection and inflammation serve as a host defense mechanism since microorganisms such as siderophilic bacteria and malarial parasites (*Plasmodium* sp.) are unable to thrive at low iron levels [1]. Iron also serves as a regulator of the immune system. For example, iron overload can decrease the humoral immune response, attenuate phagocytosis, and affect the function of T lymphocytes [7,8]. Macrophages are responsible for iron recycling by phagocytosing old and damaged erythrocytes, followed by the release of iron in heme by heme oxygenase-1 (HO-1) [9]. Proinflammatory cytokines are produced as part of the inflammatory response during infection. Inflammatory responses are usually mediated by pathogen (PAMPs) and damage (DAMPs) -associated molecular patterns. PAMPs serve as exogenous signals coming from the microbes and alert the immune system to the presence of pathogens. In contrast, DAMPs serve as endogenous signals to the innate immune system, indicating any unscheduled cell death, microbial invasion, or stress [10]. As a consequence of cytokine-mediated signaling, iron is sequestered within cells through the modulation of the expression of iron regulatory proteins, such as divalent metal transporter 1, ferroportin 1, ferritin, and hepcidin [11]. Moreover, the iron-containing enzyme cytochrome P450 has a role in the synthesis of steroid hormones and bile acids and the detoxification of toxic chemicals [12]. Iron–sulfur clusters are crucial parts of many enzymes mandatory for redox reactions involved in respiration and ATP generation [13]. 

In normal cells, excess iron is toxic and can induce oxidative stress that causes DNA damage. Oxidative stress, via the activation of mitogen-activated protein kinase (MAPK) and NF-kappa B (NF-κB)-associated signal transduction pathways, can regulate cell growth and cellular proliferation. In the mitochondria, reactive oxygen species (ROS) such as superoxide (O_2_^•−^) and hydrogen peroxide (H_2_O_2_) are produced from oxygen [7]. Increased iron levels in macrophages also lead to ROS-mediated damage to intracellular systems [14]. 

### 1.2. Iron Overload and Its Implication on Breast Cancer

**Figure 2:**
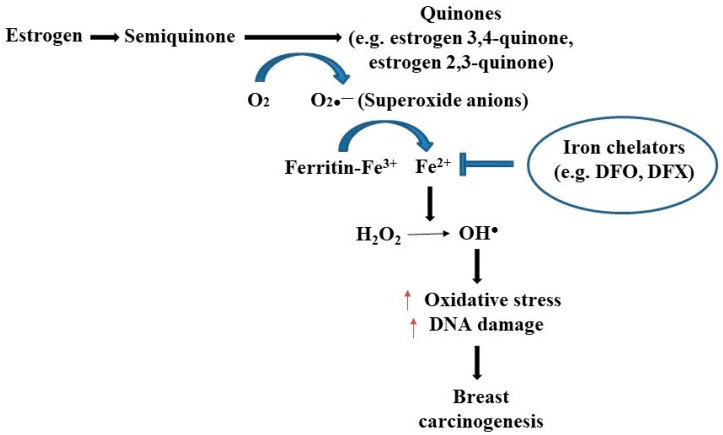
The relationship between estrogen and iron in breast cancer development. Superoxide radicals are formed during the redox cycling of estrogens to semiquinones and quinones, which reduce ferritin bound Fe^3+^ to Fe^2+^, thus releasing iron from ferritin storage sites. This free iron is responsible for creating oxidative DNA damage and breast carcinogenesis by creating hydroxyl free radical (OH.) from H_2_O_2_. The use of iron chelators like deferoxamine (DFO) and deferasirox (DFX) may halt the generation of oxidative stress followed by breast carcinogenesis [21,27].

Iron overload due to increased absorption or other disorders can be detrimental and may lead to pathological conditions that include diabetes, cardiomyopathy, neurological disorders, and various types of human cancers such as colorectal, lung, and breast carcinomas [14,15]. In the case of iron overload, the capacity of transferrin to bind iron becomes saturated, causing iron to accumulate as nontransferrin-bound iron (NTBI). The accumulation of NTBI may have significant health hazards as NTBI has the ability to catalyze free-radical reactions [16]. In this way, iron overload may predispose to oxidant-mediated breast carcinogenesis [17,18]. In breast cancer, the iron-binding as well as iron-transporting proteins are often dysregulated. For example, hepcidin is found to downregulate ferroportin levels by the post-transcriptional modification of the later. The higher hepcidin and hence lower ferroportin levels, i.e., a high hepcidin/ferroportin ratio, eventually leads to an increase in ferritin expression and consequent iron overload [19,20]. Transferrin levels were also found to be altered in breast cancer cases [8,21,22]. Elevated concentrations of estrogen in breast tissue, which disrupts intracellular iron metabolism, results in excess iron that can initiate the generation of superoxide anions and the conversion of ferritin-bound Fe^3+^ to Fe^2+^, leading to estrogen-induced oxidative stress on nucleic acids followed by carcinogenesis (Figure 2) [23,24,25]. In addition, higher levels of free iron combined with other risk factors such as genotoxic metabolites of estradiol, alcohol consumption, and ionizing radiation can increase the likelihood of breast carcinogenesis [26]. Importantly, iron overload renders postmenopausal women more susceptible to breast cancer development by initiating and promoting oxidative stress [25,27]. A recent study has shown that an elevated iron levels within the inflammatory microenvironment of breast tissue may contribute to the progression and metastasis of breast cancer [28]. 

Heme iron, exclusively obtained in animal flesh, is found to have a more damaging effect on human health. A systematic review and meta-analysis by Chang and collaborators (2019) [21] showed a significant association between intake of heme iron and increased risk of breast cancer with a pooled relative risk (RR) of 1.12 (*P*_heterogeneity_ = 0.15; 95% confidence interval (CI): 1.04–1.22). Each 1-mg/day increase of heme iron was also significantly associated with an 8% increased risk of breast cancer with a pooled RR = 1.08 (*P*_nonlinearity_ = 0.41 and 0.46 for dietary and total iron, respectively; 95% CI: 1.002–1.17). Additionally, heme iron intake showed a marginally stronger association with premenopausal (pooled RR = 1.21, *P*_heterogeneity_ = 0.05, 95% CI: 0.97–1.51) breast cancer when compared with postmenopausal (pooled RR = 1.08, *P*_heterogeneity_ = 0.28, 95% CI: 0.99–1.18) cancer. However, significance was not achieved between the groups. 

### 1.3. Breast Cancer and Organotrophic Metastasis

Breast cancer has emerged as a prominent global health concern because of its prevalence among women. According to data from the International Agency for Research on Cancer (IARC), 2.3 million new cases of female breast cancer have been reported with a death rate that is also higher than other cancers. With the advancement of science and technology, early diagnosis and effective treatment strategies have been shown to combat this disease. However, 5–10% of breast cancer patients at their early diagnosis are found to have metastatic disease, and 20–30% of patients have an increased chance of breast cancer recurrence over time [29,30].

There are several risk factors that can contribute to breast cancer. These include ethnicity; hormones; and reproductive, genetic, and environmental factors as well as lifestyle factors [31,32]. Research focusing on the genetics of breast cancer has found that cell proliferation-governing genes such as TP53, MYC, RB1, JUN, BRCA1, BRCA2, and CDK2A are associated with this disease [32,33]. Morphological and biological studies on breast cancer cells have also revealed that the tumors are heterogeneous in nature, hence, the differentiation in clinical symptoms and treatment strategies that are observed [34].

**Figure 3:**
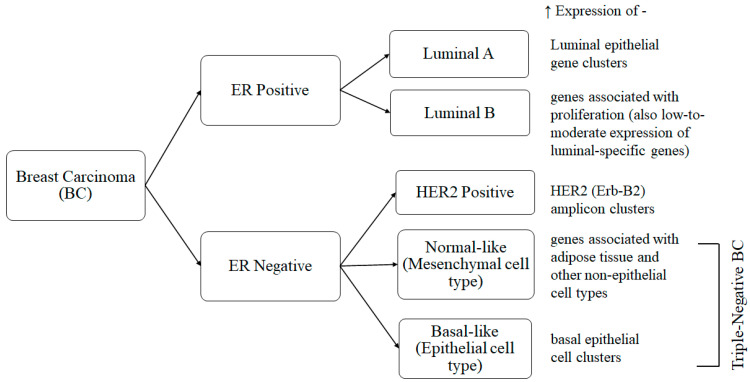
Breast carcinoma intrinsic molecular subtypes. All these subtypes (2 ER positive and 3 ER negative, of which 2 are triple-negative) are characterized by higher expressions of different subsets of genes having distinct cellular phenotypes [35,36].

Distinct cellular subtypes of breast cancer, according to latest intrinsic molecular subtyping based on gene expression profiling [35], are presented in Figure 3. All of these subtypes can metastasize from the primary tumor to other target organs. This process, termed ‘organotrophic metastasis’, depends on the tumor subtype and the microenvironment of the tumor and the other target organs. This metastasis can affect bone, liver, and lungs as well as remote lymphatic glands. The prediction of biological indication, the development of therapeutic strategies, and, ultimately, ensuring patient survival depend on an understanding of organotrophic metastasis. A pre-metastatic niche (PMN) is created when the host microenvironment adapts to changes in order to facilitate tumor growth. Moreover, this interaction of tumor cells with distant tissues also involves the host’s extracellular matrix (ECM) [33].

### 1.4. Global Trends in Breast Cancer Rates

According to the World Health Organization (WHO), breast cancer was the leading cause of mortality (a total of 10 million deaths in 2020) with a 2.3 million new cases [37]. Female breast cancer has exceeded lung cancer as the most frequently detected cancer, with an estimation of 2.3 million new cases (11.7%). The global breast cancer mortality is 685,000 [30].

The incidence rate of breast cancer is documented to be 20 times greater in higher-income countries compared with low- to middle-income countries. During the year 2016, there were approximately 719,000 cases of breast cancer in high-income countries compared with the 37,000 cases reported in the low- to middle-income countries [38]. This study used data from 1990 to 2016, comprising 195 countries that were categorized into five Sustainable Development Index (SDI) levels. However, women in higher-income countries have access to increased mammographic screening, which increases the rate of early cancer detection. The National Comprehensive Cancer Network suggests annual mammography as well as clinical breast examinations every six months [39].

North America, Australia, New Zealand, and parts of Europe have an 88% higher incidence of breast cancer than other regions, yet lower- and middle-income countries have a 17% higher mortality rate due to a lack of medical resources [30]. Women living in urban areas with better socioeconomic standing will have more treatment options in comparison with women living in rural areas. 

In South America, breast cancer is the leading cause of death, with over 30,000 cases a year, and it is considered a public health crisis. This is due to gaps in breast cancer awareness and low mammography screening [40]. In the Asia-Pacific region, countries such as China, Mongolia, and Vietnam have lower mortality rates compared with Singapore and Malaysia, which have rates comparable with the US and Australia [41]. In India, cases of breast cancer are diagnosed at a later stage. Much of the care a woman will receive in India also depends on her access to an oncologist [40]. Similarly, in Bangladesh, most patients are diagnosed when the cancer has reached an advanced stage due to the lack of awareness and inadequate access to health care facilities. Such late-stage presentation leads to increased rate of morbidity and mortality [42]. A great number of Bangladeshi women are completely ignorant to and unaware of their disease and subsequent health information due to cultural and societal norms that consider it a social disgrace to talk about sexual organs and diseases related to such organs [43]. This is also the case in Africa, where there are large rural populations with limited access to health care facilities. Many communities in South Africa attach a social stigma to breast cancer. Because of the strong community ties in many rural areas, screening, diagnosis, and treatment are often community decisions [40]. This is significant because from the 1990s to the 2010s, sub-Saharan Africa saw a rapid increase in breast cancer rates and mortality [30].

### 1.5. General and Targeted Therapies of Breast Cancer

The treatment options (Table 1) for breast cancer are commonly dependent on the presence of tumor, lymph node involvement, metastasis, staging, histological grade, hormone receptor status, ERBB2 (formerly HER2 or HER2/neu) overexpression, and menopausal status. Different groups of antineoplastic agents are used for treating breast or other cancers. These include alkylating agents, antimetabolites, cytotoxic antibiotics, and mitotic inhibitors. The categories of alkylating agents include nitrogen mustards, nitrosoureas, alkylsulfonates and platinum coordination complexes. Methotrexate and 5-flurouracil are antimetabolites that are widely used for the treatment of cancers. Several anthracycline antibiotics (doxorubicin, bleomycin, dactinomycin, etc.) are also used as anticancer drugs. Taxanes such as docetaxel and paclitaxel are the mitotic inhibitors most often used to treat breast and other cancers [44].

Early-stage breast cancer treated by radiation therapy followed by breast-conserving surgery has the highest success rates in women [45]. Many countries rarely treat postmenopausal women with endocrine therapy because of its undesirable side effects. However, in the USA, the hormone therapy rate is higher [46]. Randomized controlled clinical trials have shown that the use of tamoxifen or aromatase inhibitors is beneficial for women who have a high risk of breast cancer [47].

Targeted cancer therapies are used when breast tumor cells exhibit particular characteristics, for example, a gene or a factor that allows the cancer cells to grow in a rapid or irregular way [48]. These therapies are less likely to damage healthy cells in comparison with conventional chemotherapy. Several targeted therapeutic agents have already shown their effectiveness and are widely used as targeted treatments for the treatment of breast cancer [49,50]. The following medications have already shown their effectiveness as a targeted treatments:

1. Inhibitors Targeting Protein Kinase B (Akt)/Phosphatidylinositol 3-Kinase (PI3K)/Mammalian Target of Rapamycin (mTOR) Pathway
2. Cyclin-Dependent Kinases 4 and 6 Inhibitors (CDK 4/6 Inhibitors)
3. Poly (ADP ribose) Polymerase (PARP) Inhibitors
4. Tyrosine Kinase Inhibitors
5. Vascular Endothelial Growth Factor-A Inhibitors

The current multimodal approach employed for breast cancer treatment has undoubtedly improved the overall survival of patients and has greatly improved their quality of life [53]. However, be it conventional chemotherapy, targeted therapy, or immunotherapy, none are free from limitations. The conventional chemotherapeutic agents, including platins, anthracyclins, and taxanes, apart from causing blood and bone marrow toxicities, can also give rise to various types of cardiotoxicity, thus limiting their use, particularly in patients with cardiac dysfunction [54]. Trastuzumab is only effective for the treatment of ERBB2-overexpressing tumors, which are a minority of breast cancer cases [55]. Targeted cancer therapy including tyrosine kinase inhibitors (e.g., gefetinib, erlotinib) and aromatase inhibitors (e.g., anastrozole, letrozole and exemestane) have shown toxicities and low clinical efficacy, thus necessitating the use of combination therapy [56]. Cancer immunotherapies, including immune checkpoint inhibitors such as pembrolizumab, ipilimumab, and atezolizumab, only demonstrate clinical usefulness for a few specific types of breast cancer [53]. 

Considering the limitations of current therapeutic approaches, it is important to develop alternative therapies that would give additional benefit to breast cancer patients without serious toxic side effects. Therefore, in this review, we present iron chelators as an effective alternative treatment modality for breast cancer alongside conventional chemotherapeutic agents.

## 2. A Summary of a Search of Literatures/Records 

**Figure 4:**
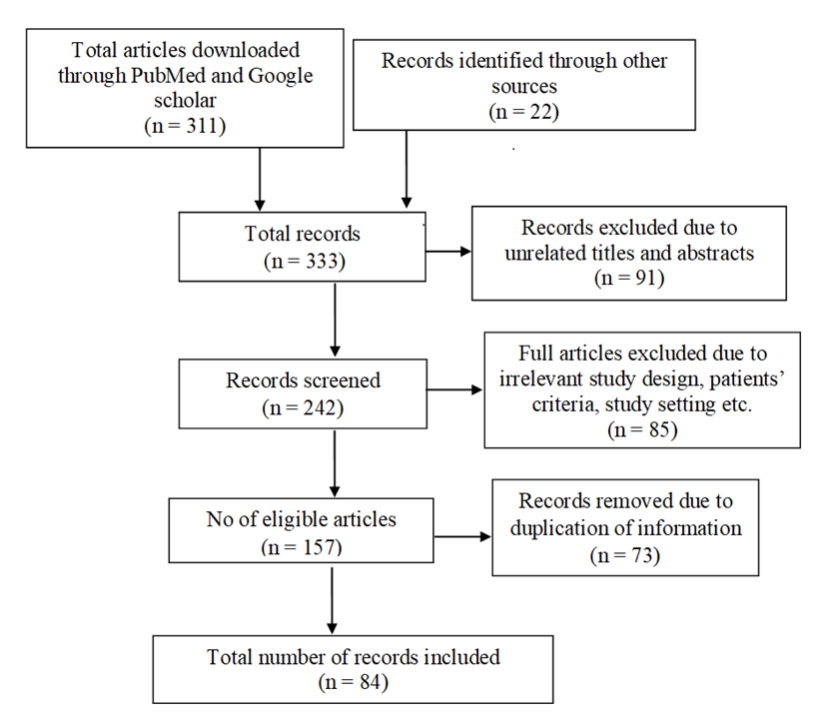
A summary of the literature search.

The PubMed and Google Scholar search parameters were set to the last 20 years. The search was performed from March 2021 to May 2022, with the last search being performed on 28 May 2022. The strategy involved searching the databases using the following Medical Subject Heading (MeSH) terms: ‘breast cancer’, ‘invasive breast cancer’, ‘targeted therapy’, ‘cancer stages’, ‘cancer type’, ‘primary therapy’, ‘iron overload’, ‘iron chelator’, ‘deferoxamine’, ‘deferasirox’, ‘NF-κB’, and ‘ROS’. A total of 333 records were identified. After the removal of 249 records due to irrelevant study design and duplication of information, 84 papers were included for this review (Figure 4). The inclusion criteria for the records were original articles, review articles, and websites focused on breast cancer diagnosis, prognosis, treatment, recent discoveries, and clinical case studies. 

## 3. Iron Chelation in Treating Cancers

Neoplastic/cancer cells have a higher requirement for iron due to their continuous and rapid proliferation [57]. In order to do that, the cancer cells alter iron homeostasis for encountering this excessive demand [4]. Compared with the untransformed cells, cancer cells require an increased amount of iron for their metabolic functions. They use several mechanisms to maintain or increase the amount of iron. The mechanisms include (i) the upregulation of transferrin receptors which bind with the transferrin binding iron (ferric iron), (ii) the transportation of ferrous iron into cytosol via divalent metal-ion transporter, which joins the labile iron pool, and (iii) the decreased expression of ferroportin, which is associated with the anaplasia of the cancer tissues with the reduction of metastasis-free survival of patients with breast cancers [19]. This excess iron catalyzes the synthesis of ROS, which may result in DNA damage, the subsequent loss or inactivation of tumor suppressor genes, and/or the activation of oncogenes [11]. ROS contributes to the activation of NF-κB, which is responsible for regulating inflammatory mediators such as TNFα [58]. Additionally, ferritin has a critical role in the mechanism of tumor progression, and the suppression of ferritin levels thus can kill cancer cells and disrupt the tumor microenvironment [59].

The increased iron environment safeguards cancer cells from the cytolysis by natural killer cells, and thereby, apoptosis of the cancer cells is protected. Jiang and Elliott (2017) [60] reported that an increased iron environment antagonizes NO- and TNFα-associated cytotoxicity. Ferritin expression in breast cancer cells is also upregulated. This process can be reversed if the iron concentration is decreased in the cancer cells. In their experiment, natural killer cell lines (NK-92MI) were used to co-culture human breast cancer cell lines (MCF-7 and MDA-MB-231) to determine their cytolysis. Increased NO and TNFα were found during the co-culturing of human breast cancer cell lines with the NK-92MI. Cytolysis in these cell lines was inhibited after the addition of more iron. 

Therefore, iron chelation could be a promising approach to reducing the generation of ROS and its associated carcinogenicity. Iron chelators such as deferoxamine (DFO) and deferasirox (DFX) have shown potential antineoplastic activity by limiting iron bioavailability to the malignant cells [9,61,62]. Iron chelators may exert their anticancer potential by depleting intracellular iron levels, inhibiting DNA synthesis, promoting cancer cell apoptosis, or causing oxidative stress in the tumor microenvironment [63,64,65]. Furthermore, the reduction of intracellular iron by iron chelation is able to sensitize breast cancer cells to chemotherapeutic agents [66]. A recent study in iron-deficient rats reported a significant reduction in mammary cancer incidence [67]. The effect of iron withdrawal by chelation has been investigated in triple-negative (TNBC) and hormone-receptor-positive breast cancer cell lines [68]. Breast cancer cell proliferation was inhibited by iron chelation; however, iron removal also produces hypoxia and angiogenesis that may promote tumor progression. The combination of eribulin and iron chelation inhibited the growth of breast cancer xenografts in mice to a greater extent than monotherapy with eribulin or the iron chelator. A synergistic effect of DFX is also observed with other chemotherapeutic agents like cisplatin, carboplatin, and doxorubicin, resulting in the inhibition of cell proliferation, the induction of apoptosis, and the autophagy of TNBC cells [69]. Another study demonstrated a disruption of intracellular iron homeostasis when breast cancer cells were treated with a high dose of the iron chelator DFO [70], resulting in a reduction in cell viability and growth. In this regard, DFO inhibits DNA synthesis in several cancer cell lines [9]. High-dose DFO also induces apoptosis in both metastatic and nonmetastatic breast cancer cell lines [71]. It has also been reported that the treatment with DFO increased the NK-92MI cytolysis in human breast cancer cells [60]. Importantly, monotherapy with desferal (DFO) at physiologically achievable concentrations significantly inhibits breast cancer cell growth in vitro, as well as the breast tumor xenograft growth in mice. 

Orally active DFX is superior to DFO because of its longer plasma half-life and higher affinity for iron [72]. Due to its effectiveness and low toxicity, a number of preclinical and clinical studies have investigated the effectiveness of iron chelation with DFX in the treatment of cancer [61,72,73]. DFX inhibits DNA synthesis and cellular metabolism, induces DNA fragmentation, and blocks cell cycle progression during S-phase, as well as suppressing ROS generation [74,75]. Although there are limited clinical data on the use of DFX, it is a safe and effective iron chelator and could be a potential chemotherapeutic candidate [76]. Both DFO and DFX reduce levels of ROS, NF-κB, and ferritin in association with a reduction in the labile iron pool [9,77]. Therefore, iron chelators may have potential benefits in decreasing the uptake of iron by breast cancer cells along with other cancer cell types. However, as iron withdrawal tends to inhibit intracellular ROS production, it is important to exercise caution when using iron chelators in combination with treatment modalities that rely on the ROS-mediated destruction of cancer cells.

## 4. Dual Roles of Iron in Cancer

The potential use of iron modulation as a treatment for breast cancer, although promising, is not at all straightforward. Iron withdrawal via selective chelation and the subsequent decrease in intracellular iron inhibits cancer cell growth and spread to distant parts of the body, as well as inducing cancer cell death by apoptosis, since the increased uptake and reduced export of iron are a requirement for the rapid division and metastasis of cancer cells, as well as their survival [78]. On the other hand, intracellular iron levels that allow for the generation of toxic levels of ROS determine whether a cancer cell is susceptible to a form of iron-dependent regulated cell death known as ferroptosis [79]. Moreover, under some conditions, iron deficiency can promote cancer cell invasion and migration by mimicking hypoxia [80], and certain types of ROS can also promote metastasis [81]. The subcellular localization of iron within cancer cells may contribute to the diverse and sometimes conflicting effects of iron modulation.

The primary route for the transport of iron into cancer cells is via the endocytosis of transferrin receptors that have bound iron-carrying transferrin. The acidic environment of the endosomes causes iron to dissociate from transferrin followed by divalent metal ion transporter 1-mediated export into the intracellular labile iron pool, where it is available for use, storage, or export by ferroportin. Intracellular iron is utilized within the mitochondria, nucleus, and cytoplasm. Mitochondrial iron is required for the synthesis of heme and Fe-S clusters involved in ATP production [82]. In the cytoplasm and nucleus, iron is a critical cofactor for the activity of many enzymes, including deoxyhypusine hydroxylase and ribonucleotide reductase [83,84]. Excess iron is safely stored in different cellular compartments, primarily in the form of ferritin, until released as required. Intracellular iron that exceeds the cell’s storage capacity leads to the Fenton reaction-induced generation of ROS that can promote cancer-causing mutations, leading to cancer progression or, if ROS levels are high enough, cytotoxicity. Restricting the availability of extracellular iron via chelation causes a reduction in the intracellular labile iron pool, leading to mitochondrial dysfunction and the impaired activity of enzymes involved in cancer cell survival, growth, and metastasis. Iron deficiency will also inhibit ROS generation by the Fenton reaction, thereby interfering with carcinogenesis and cancer progression, although at the same time rendering the cancer cell less susceptible to ferroptosis. In addition, iron deficiency can promote neo-vascularization due to hypoxia-inducible factor 1a (HIF1a) expression and stabilization [80], as well as causing the production of mitochondrial ROS that enhances cancer cell invasion and migration [81]. In this way, limiting iron availability to cancer cells has the potential to promote metastasis. 

Although the use of iron chelators to restrict cancer cells’ utilization of iron clearly shows potential as a treatment for breast cancer and other cancers, a case can also be made for iron-loading to induce ferroptosis in cancer cells. A better understanding of altered iron metabolism in breast cancer cells is needed to effectively implement iron modulation as a strategy for cancer treatment. 

## 5. Conclusions

To decrease breast cancer morbidity and mortality, proper management and treatment should be provided to patients following early diagnosis. Conventional and targeted treatment of breast cancer may reduce patient morbidity; however, the cost of breast cancer management can be very high, particularly with the targeted treatments. The expense of breast cancer treatment serves as a barrier to pursuing medical intervention, particularly in patients who live in developing countries. It is therefore important to pursue a simple strategy for treating patients with breast cancer. Iron is a precipitating factor in breast cancer cell growth and multiplication, which may lead to metastasis. Excess iron along with the disruption of intracellular iron metabolism by estrogen also plays a major role in the development and progression of breast cancer. Iron withdrawal may have the benefit of preventing cancer cell growth, as well as reducing inflammation by suppressing the generation of ROS. Therefore, iron chelators can have a major role in addition to other therapeutic approaches for the management of breast cancer. However, iron chelation can only be considered as a therapeutic strategy after the confirmation of its efficacy and safety in randomized controlled trials.

## Figures and Tables

**Table 1:** Treatment Options for Breast Cancer.

Cancer Stage		Stage 0		Stage I & Stage II	Stage III		Stage IV	Reference(s)
Cancer Type		Lobular carcinoma (in situ).	Ductal carcinoma (in situ).	Early stage invasive carcinoma.	Locally advanced noninflammatory carcinoma.	Locally advanced inflammatory carcinoma.	Initial or recurrent metastatic condition.	[45]
Primary Therapy		NT or PT + TXF.	BCS&RT	BCS & RT.	Ind. Chemo. + BCS. & RT.	Ind. chemo. + mastecto my & RT.	RT.	[45,51]
Adjuvant Therapy	Negative hormone receptor			Chemotherapy.	Ind. chemo. & ET.		Chemotherapy.	[45]
	Positive hormone receptor			Chemotherapy & ET.	Ind. chemo. & ET.		ET+/chemotherapy.	[45,51]
	ERBB2 Overexpression			Chemotherapy. &TRA (Herceptin).	Ind. Chemo. & TRA.		TRA+/chemotherapy.	[45,52]

Here, NT = No Treatment; PT = Prophylaxis Treatment; TXF = Tamoxifen; BCS = Breast conserving surgery; RT = Radiation Therapy; Ind. Chemo. = Induction Chemotherapy; ET = Endocrine Therapy; TRA = Trastuzumab; ERBB2 = Erythroblastic Oncogene-B 2, a gene isolated from avian genome.

## Data Availability

Not applicable.

## Abbreviations

WHO: World Health Organization; IARC: International Agency for Research on Cancer; NTBI: Nontransferrin-bound iron; ROS: Reactive oxygen species; PAMPs: Pathogen-associated molecular patterns; DAMPs: Damage-associated molecular patterns; MAPK: Mitogen-activated protein kinase; NF-κB: Nuclear factor kappa-light-chain-enhancer of activated B cells; TP53: Tumor protein 53; BRCA1: Breast cancer gene 1; BRCA2: Breast cancer gene 2; CDK2A: Cyclin-dependent kinase inhibitor 2A; PMN: Pre-metastatic niche; ECM: Extracellular matrix; SDI: Sustainable Development Index; ERBB2: Erythroblastic oncogene B; HER2: Human epidermal growth factor receptor 2; PI3K: Phosphatidylinositol 3-Kinase; mTOR: Mammalian target of rapamycin; CDK 4/6: Cyclin-dependent kinases 4 and 6; PARPs: Poly ADP ribose polymerase; TNBC: Triple-negative breast cancer; DFO: Deferoxamine; DFX: Deferasirox; RR = relative risk.

## References

[1] Ganz T., Nemeth E. (2015). Iron homeostasis in host defence and inflammation. Nat. Rev. Immunol..

[2] Jiang Y., Li C., Wu Q., An P., Huang L., Wang J., Chen C., Chen X., Zhang F., Ma L. (2019). Iron-dependent histone 3 lysine 9 demethylation controls B cell proliferation and humoral immune responses. Nat. Commun..

[3] Wallace D.F. (2019). Regulation of Folate Homeostasis. Montreal, Canada, 15–20 June 1986.

[4] Saha P., Yeoh B.S., Xiao X., Golonka R.M., Kumarasamy S., Vijay-Kumar M. (2019). Enterobactin, an iron chelating bacterial siderophore, arrests cancer cell proliferation. Biochem. Pharmacol..

[5] Abbaspour N., Hurrell R., Kelishadi R. (2014). Review on iron and its importance for human health. J. Res. Med. Sci..

[6] Knutson M.D. (2017). Iron transport proteins: Gateways of cellular and systemic iron homeostasis. J. Biol. Chem..

[7] Lamy P.J., Durigova A., Jacot W. (2014). Iron homeostasis and anemia markers in early breast cancer iron and breast cancer. Clin. Chim. Acta.

[8] Walker J., Walker S.M. (2000). Review: Effects of iron overload on the immune system. Ann. Clin. Lab. Sci..

[9] Hatcher H.C., Singh R.N., Torti F.M., Torti S.V. (2009). Synthetic and natural iron chelators: Therapeutic potential and clinical use. Futur. Med. Chem..

[10] Tang D., Kang R., Coyne C.B., Zeh H.J., Lotze M.T. (2012). PAMPs and DAMPs: Signal 0s that spur autophagy and immunity. Immunol. Rev..

[11] Lehmann C., Islam S., Jarosch S., Zhou J., Hoskin D., Greenshields A., Al-Banna N., Sharawy N., Sczcesniak A., Kelly M. (2015). The Utility of Iron Chelators in the Management of Inflammatory Disorders. Mediat. Inflamm..

[12] Yu Y., Gutierrez E., Kovacevic Z., Saletta F., Obeidy P., Rahmanto Y.S., Richardson D.R. (2012). Iron Chelators for the Treatment of Cancer. Curr. Med. Chem..

[13] Rouault T.A., Tong W.H. (2008). Iron–sulfur cluster biogenesis and human disease. Trends Genet..

[14] Ward D.M., Cloonan S.M. (2019). Mitochondrial Iron in Human Health and Disease. Annu. Rev. Physiol..

[15] Menshawey R., Menshawey E., Alserr A.H.K., Abdelmassih A.F. (2020). Low iron mitigates viral survival: Insights from evolution, genetics, and pandemics—a review of current hypothesis. Egypt. J. Med Hum. Genet..

[16] Hershko C. (2007). Iron loading and its clinical implications. Am. J. Hematol..

[17] Bae Y.-J., Yeon J.-Y., Sung C.-J., Kim H.-S., Sung M.-K. (2009). Dietary Intake and Serum Levels of Iron in Relation to Oxidative Stress in Breast Cancer Patients. J. Clin. Biochem. Nutr..

[18] Fonseca-Nunes A., Jakszyn P., Agudo A. (2014). Iron and Cancer Risk—A Systematic Review and Meta-analysis of the Epidemiological Evidence. Cancer Epidemiol. Biomark. Prev..

[19] Pinnix Z.K., Miller L.D., Wang W., D’Agostino R., Kute T., Willingham C., Hatcher H., Tesfay L., Sui G., Di X. (2010). Ferroportin and Iron Regulation in Breast Cancer Progression and Prognosis. Sci. Transl. Med..

[20] Zhang S., Chen Y., Guo W., Yuan L., Zhang D., Xu Y., Nemeth E., Ganz T., Liu S. (2014). Disordered hepcidin–ferroportin signaling promotes breast cancer growth. Cell. Signal..

[21] Chang V.C., Cotterchio M., Khoo E. (2019). Iron intake, body iron status, and risk of breast cancer: A systematic review and meta-analysis. BMC Cancer.

[22] Mustafa R.M.A., Husain N.E.O.S.A. (2017). Changes in Serum Iron, Total Iron Binding Capacity and Transferrin Saturation Percent in Sudanese Females Newly Diagnosed with Breast Cancer at Khartoum Oncology Hospital: A case- control study. Sudan J. Med Sci..

[23] Bajbouj K., Shafarin J., Abdalla M.Y., Ahmad I., Hamad M. (2017). Estrogen-induced disruption of intracellular iron metabolism leads to oxidative stress, membrane damage, and cell cycle arrest in MCF-7 cells. Tumor Biol..

[24] Cavalieri E., Chakravarti D., Guttenplan J., Hart E., Ingle J., Jankowiak R., Muti P., Rogan E., Russo J., Santen R. (2006). Catechol estrogen quinones as initiators of breast and other human cancers: Implications for biomarkers of susceptibility and cancer prevention. Biochim. Biophys. Acta.

[25] Jian J., Yang Q., Dai J., Eckard J., Axelrod D., Smith J., Huang X. (2011). Effects of iron deficiency and iron overload on angiogenesis and oxidative stress—A potential dual role for iron in breast cancer. Free Radic. Biol. Med..

[26] Kabat G.C., Rohan T.E. (2007). Does excess iron play a role in breast carcinogenesis? An unresolved hypothesis. Cancer Causes Control.

[27] Huang X. (2008). Does iron have a role in breast cancer?. Lancet Oncol..

[28] Cheng M., Liu P., Xu L.X. (2020). Iron promotes breast cancer cell migration via IL-6/JAK2/STAT3 signaling pathways in a paracrine or autocrine IL-6-rich inflammatory environment. J. Inorg. Biochem..

[29] Claessens A.K.M., Ibragimova K.I.E., Geurts S.M.E., Bos M.E.M.M., Erdkamp F.L.G., Tjan-Heijnen V.C.G. (2020). The role of chemotherapy in treatment of advanced breast cancer: An overview for clinical practice. Crit. Rev. Oncol./Hematol..

[30] Sung H., Ferlay J., Siegel R.L., Laversanne M., Soerjomataram I., Jemal A., Bray F. (2021). Global Cancer Statistics 2020: GLOBOCAN Estimates of Incidence and Mortality Worldwide for 36 Cancers in 185 Countries. CA Cancer J. Clin..

[31] Brody J.G., Moysich K.B., Humblet O., Attfield K.R., Beehler G.P., Rudel R.A. (2007). Environmental pollutants and breast cancer: Epidemiologic studies. Cancer.

[32] Momenimovahed Z., Salehiniya H. (2019). Epidemiological characteristics of and risk factors for breast cancer in the world. Breast Cancer Targets Ther..

[33] Yazici H., Akin B. (2020). Molecular Genetics of Metastatic Breast Cancer. Tumor Progress. Metastasis.

[34] Tsang J.Y.S., Tse G.M. (2020). Molecular Classification of Breast Cancer. Adv. Anat. Pathol..

[35] Lee Y.-M., Oh M.H., Go J.-H., Han K., Choi S.-Y. (2020). Molecular subtypes of triple-negative breast cancer: Understanding of subtype categories and clinical implication. Genes Genom..

[36] Sørlie T., Perou C.M., Tibshirani R., Aas T., Geisler S., Johnsen H., Hastie T., Eisen M.B., Van De Rijn M., Jeffrey S.S. (2001). Gene expression patterns of breast carcinomas distinguish tumor subclasses with clinical implications. Proc. Natl. Acad. Sci. USA.

[37] WHO (2022). Cancer. https://www.who.int/news-room/fact-sheets/detail/cancer.

[38] Hu K., Ding P., Wu Y., Tian W., Pan T., Zhang S. (2019). Global patterns and trends in the breast cancer incidence and mortality according to sociodemographic indices: An observational study based on the global burden of diseases. BMJ Open.

[39] Gradishar W.J., Anderson B.O., Abraham J., Aft R., Agnese D., Allison K.H., Blair S.L., Burstein H.J., Dang C., Elias A.D. (2020). NCCN Clinical Guidelines Breast Cancer (Version 5.2020): Invasive Breast Cancer.

[40] Collyar D.E. (2001). Breast cancer: A global perspective. J. Clin. Oncol..

[41] Binns C., Low W.Y., Lee M.K. (2013). Breast cancer: An increasing public health problem in the Asia pacific region. Asia-Pac. J. Public Health.

[42] Islam R.M., Bell R.J., Billah B., Hossain M.B., Davis S.R. (2015). Awareness of breast cancer and barriers to breast screening uptake in Bangladesh: A population based survey. Maturitas.

[43] Ahmed S., Sayeed A., Mallick T., Syfuddin H.M. (2020). Knowledge and Practices on Breast Cancer among Bangladeshi Female University Students: A Cross-sectional Study. Asian Pac. J. Cancer Care.

[44] Burguin A., Diorio C., Durocher F. (2021). Breast Cancer Treatments: Updates and New Challenges. J. Pers. Med..

[45] Moo T.-A., Sanford R., Dang C., Morrow M. (2018). Overview of Breast Cancer Therapy. PET Clin..

[46] van Seijen M., Lips E.H., Thompson A.M., Nik-Zainal S., Futreal A., Hwang E.S., Verschuur E., Lane J., Jonkers J., Rea D.W. (2019). Ductal carcinoma in situ: To treat or not to treat, that is the question. Br. J. Cancer.

[47] Wen H.Y., Brogi E. (2018). Lobular Carcinoma In Situ. Surg. Pathol. Clin..

[48] Cooper G.M. (2000). The Development and Causes of Cancer. The Cell: A Molecular Approach.

[49] Masoud V., Pagès G. (2017). Targeted therapies in breast cancer: New challenges to fight against resistance. World J. Clin. Oncol..

[50] Ross J.S., Schenkein D.P., Pietrusko R., Rolfe M., Linette G.P., Stec J., Stagliano N.E., Ginsburg G.S., Symmans W.F., Pusztai L. (2004). Targeted Therapies for Cancer 2004. Am. J. Clin. Pathol..

[51] Maughan K.L., Lutterbie M.A., Ham P.S. (2010). Treatment of breast cancer. Am. Fam. Physician.

[52] Mehata A.K., Bharti S., Singh P., Viswanadh M.K., Kumari L., Agrawal P., Singh S., Koch B., Muthu M.S. (2018). Trastuzumab decorated TPGS-g-chitosan nanoparticles for targeted breast cancer therapy. Colloids Surf. B Biointerfaces.

[53] Tokumaru Y., Joyce D., Takabe K. (2019). Current status and limitations of immunotherapy for breast cancer. Surgery.

[54] Bird B.R.H., Swain S.M. (2008). Cardiac Toxicity in Breast Cancer Survivors: Review of Potential Cardiac Problems. Clin. Cancer Res..

[55] Stebbing J., Copson E.O.S. (2000). Herceptin (trastuzamab) in advanced breast cancer. Cancer Treat. Rev..

[56] Curigliano G., Criscitiello C. (2014). Successes and limitations of targeted cancer therapy in breast cancer. Prog. Tumor Res..

[57] Bedford M.R., Ford S.J., Horniblow R.D., Iqbal T.H., Tselepis C. (2013). Iron Chelation in the Treatment of Cancer: A New Role for Deferasirox?. J. Clin. Pharmacol..

[58] Taniguchi K., Karin M. (2018). NF-κB, inflammation, immunity and cancer: Coming of age. Nat. Rev. Immunol..

[59] Alkhateeb A.A., Connor J.R. (2013). The significance of ferritin in cancer: Anti-oxidation, inflammation and tumorigenesis. Biochim. Biophys. Acta.

[60] Jiang X.-P., Elliott R.L. (2017). Decreased Iron in Cancer Cells and Their Microenvironment Improves Cytolysis of Breast Cancer Cells by Natural Killer Cells. Anticancer Res..

[61] Ford S., Obeidy P., Lovejoy D., Bedford M., Nichols L., Chadwick C., Tucker O., Lui G., Kalinowski D., Jansson P. (2013). Deferasirox (ICL670A) effectively inhibits oesophageal cancer growth in vitro and in vivo. Br. J. Pharmacol..

[62] Kontoghiorghe C.N., Kontoghiorghes G.J. (2016). Efficacy and safety of iron-chelation therapy with deferoxamine, deferiprone, and deferasirox for the treatment of iron-loaded patients with non-transfusion-dependent thalassemia syndromes. Drug Des. Dev. Ther..

[63] Buss J.L., Greene B.T., Turner J., Torti F.M., Torti S.V. (2004). Iron Chelators in Cancer Chemotherapy. Curr. Top. Med. Chem..

[64] Coombs R.P.M., Grant T., Greenshields A.L., Arsenault D.J., Holbein B.E., Hoskin D.W. (2015). Inhibitory effect of iron withdrawal by chelation on the growth of human and murine mammary carcinoma and fibrosarcoma cells. Exp. Mol. Pathol..

[65] Jung M., Mertens C., Tomat E., Brüne B. (2019). Iron as a Central Player and Promising Target in Cancer Progression. Int. J. Mol. Sci..

[66] Greenshields A.L., Coombs M.R.P., Fernando W., Holbein B.E., Hoskin D.W. (2019). DIBI, a novel 3-hydroxypyridin-4-one chelator iron-binding polymer, inhibits breast cancer cell growth and functions as a chemosensitizer by promoting S-phase DNA damage. BioMetals.

[67] Thompson H.J., Neil E.S., McGinley J.N. (2021). Pre-Clinical Insights into the Iron and Breast Cancer Hypothesis. Biomedicines.

[68] Goto W., Kashiwagi S., Asano Y., Takada K., Morisaki T., Takahashi K., Fujita H., Shibutani M., Amano R., Takashima T. (2020). Inhibitory effects of iron depletion plus eribulin on the breast cancer microenvironment. BMC Cancer.

[69] Tury S., Assayag F., Bonin F., Chateau-Joubert S., Servely J.-L., Vacher S., Becette V., Caly M., Rapinat A., Gentien D. (2018). The iron chelator deferasirox synergises with chemotherapy to treat triple-negative breast cancers. J. Pathol..

[70] Bajbouj K., Shafarin J., Hamad M. (2018). High-Dose Deferoxamine Treatment Disrupts Intracellular Iron Homeostasis, Reduces Growth, and Induces Apoptosis in Metastatic and Nonmetastatic Breast Cancer Cell Lines. Technol. Cancer Res. Treat..

[71] Hoke E.M., Maylock C.A., Shacter E. (2005). Desferal inhibits breast tumor growth and does not interfere with the tumoricidal activity of doxorubicin. Free Radic. Biol. Med..

[72] Lui G.Y.L., Obeidy P., Ford S.J., Tselepis C., Sharp D.M., Jansson P.J., Kalinowski D.S., Kovacevic Z., Lovejoy D.B., Richardson D.R. (2012). The Iron Chelator, Deferasirox, as a Novel Strategy for Cancer Treatment: Oral Activity against Human Lung Tumor Xenografts and Molecular Mechanism of Action. Mol. Pharmacol..

[73] Messa E., Cilloni D., Messa F., Arruga F., Roetto A., Saglio G. (2008). Deferasirox Treatment Improved the Hemoglobin Level and Decreased Transfusion Requirements in Four Patients with the Myelodysplastic Syndrome and Primary Myelofibrosis. Acta Haematol..

[74] Chantrel-Groussard K., Gaboriau F., Pasdeloup N., Havouis R., Nick H., Pierre J.-L., Brissot P., Lescoat G. (2006). The new orally active iron chelator ICL670A exhibits a higher antiproliferative effect in human hepatocyte cultures than O-trensox. Eur. J. Pharmacol..

[75] Toyokuni S. (2009). Role of iron in carcinogenesis: Cancer as a ferrotoxic disease. Cancer Sci..

[76] Fukushima T., Kawabata H., Nakamura T., Iwao H., Nakajima A., Miki M., Sakai T., Sawaki T., Fujita Y., Tanaka M. (2011). Iron chelation therapy with deferasirox induced complete remission in a patient with chemotherapy-resistant acute monocytic leukemia. Anticancer Res..

[77] Messa E., Carturan S., Maffè C., Pautasso M., Bracco E., Roetto A., Messa F., Arruga F., Defilippi I., Rosso V. (2010). Deferasirox is a powerful NF- B inhibitor in myelodysplastic cells and in leukemia cell lines acting independently from cell iron deprivation by chelation and reactive oxygen species scavenging. Haematologica.

[78] Brown R.A.M., Richardson K.L., Kabir T.D., Trinder D., Ganss R., Leedman P.J. (2020). Altered Iron Metabolism and Impact in Cancer Biology, Metastasis, and Immunology. Front. Oncol..

[79] Dixon S.J., Stockwell B.R. (2019). The Hallmarks of Ferroptosis. Annu. Rev. Cancer Biol..

[80] Eckard J., Dai J., Wu J., Jian J., Yang Q., Chen H., Costa M., Frenkel K., Huang X. (2010). Effects of cellular iron deficiency on the formation of vascular endothelial growth factor and angiogenesis. Iron deficiency and angiogenesis. Cancer Cell Int..

[81] Prutki M., Poljak-Blazi M., Jakopovic M., Tomas D., Stipancic I., Zarkovic N. (2006). Altered iron metabolism, transferrin receptor 1 and ferritin in patients with colon cancer. Cancer Lett..

[82] Lane D.J.R., Merlot A.M., Huang M.L.H., Bae D.H., Jansson P.J., Sahni S., Kalinowski D.S., Richardson D.R. (2015). Cellular iron uptake, trafficking and metabolism: Key molecules and mechanisms and their roles in disease. Biochim. Biophys. Acta.

[83] Epis M.R., Giles K.M., Kalinowski F.C., Barker A., Cohen R.J., Leedman P.J. (2012). Regulation of Expression of Deoxyhypusine Hydroxylase (DOHH), the Enzyme That Catalyzes the Activation of eIF5A, by miR-331-3p and miR-642-5p in Prostate Cancer Cells. J. Biol. Chem..

[84] Puig S., Ramos-Alonso L., Romero A.M., Martínez-Pastor M.T. (2017). The elemental role of iron in DNA synthesis and repair. Metallomics.

